# Statins as a risk factor for diabetic retinopathy: a Mendelian randomization and cross-sectional observational study

**DOI:** 10.1186/s12967-024-05097-8

**Published:** 2024-03-22

**Authors:** Chengming Chen, Huan Zhang, Yanyan Lan, Weiming Yan, Sida Liu, Yixuan Chen, Tingke Xie, Jiayi Ning, Xiaolong Yan, Lei Shang, Jing Han

**Affiliations:** 1 Department of Ophthalmology, Tangdu Hospital, The Air Force Military Medical University, 1 Xinsi Rd, Xi’an, 710038 China; 2 Department of Ophthalmology, The 900th Hospital of Joint Logistic Support Force, PLA (Fuzong Clinical Medical College of Fujian Medical University), Fuzhou, 350025 China; 3 Department of Gastroenterology, Air Force Medical Center, The Air Force Military Medical University, Beijing, China; 4 College of Rehabilitation Medicine, Fujian University of Traditional Chinese Medicine, Fuzhou, 350122 China; 5 Department of Thoracic Surgery, Tangdu Hospital, The Air Force Military Medical University, 1 Xinsi Rd, Xi’an, 710038 China; 6 Department of Health Statistics, School of Preventive Medicine, The Air Force Military Medical University, Xi’an, 710038 China

**Keywords:** Diabetic retinopathy, Statins, HMGCR, Mendelian randomization, NHANES

## Abstract

**Background:**

Diabetic retinopathy (DR) is the foremost cause of vision loss among the global working-age population, and statins are among the most frequently prescribed drugs for lipid management in patients with DR. The exact relationship between statins and DR has not been determined. This study sought to validate the causal association between statins usage and diabetic retinopathy.

**Methods:**

The summary-data-based Mendelian randomization (SMR) method and inverse-variance-weighted Mendelian randomization (IVW-MR) were used to identify the causal relationship between statins and DR via the use of expression quantitative trait loci (eQTL) data for 3-hydroxy-3-methylglutaryl-coenzyme A reductase (HMGCR) (31,684 blood samples), low density lipoprotein cholesterol-related GWAS data (sample size: 440,546), and DR-related GWAS data (14,584 cases and 176,010 controls). Additionally, a cross-sectional observational study based on the data from the National Health and Nutrition Examination Survey (NHANES) was conducted to supplement the association between DR and statins (sample size: 106,911). The odds ratios (ORs) with corresponding 95% confidence intervals (CIs) was employed to evaluate the results.

**Results:**

Based on the results of the MR analysis, HMGCR inhibitors were causally connected with a noticeably greater incidence of DR (IVW: OR = 0.54, 95% CI [0.42, 0.69], p = 0.000002; SMR: OR = 0.66, 95% CI [0.52, 0.84], p = 0.00073). Subgroup analysis revealed that the results were not affected by the severity of DR. The sensitivity analysis revealed the stability and reliability of the MR analysis results. The results from the cross-sectional study based on NHANES also support the association between not taking statins and a decreased risk of DR (OR = 0.54, 95% CI [0.37, 0.79], p = 0.001).

**Conclusions:**

This study revealed that a significant increase in DR risk was causally related to statins use, providing novel insights into the role of statins in DR. However, further investigations are needed to verify these findings.

**Supplementary Information:**

The online version contains supplementary material available at 10.1186/s12967-024-05097-8.

## Introduction

With a dramatic rise in the number of diabetes mellitus (DM) worldwide, diabetic retinopathy (DR), a complication of DM that affects the vision of patients, is increasing the economic burden on human society and seriously affects the quality of life of diabetic retinopathy (DR) patients [1, 2]. It is estimated that the number of people with impaired glucose tolerance worldwide will reach 548 million and that the number of DR patients will increase to a staggering 160 million by 2045 [3, 4]. Currently, the clinical treatment methods for DR mainly include laser photocoagulation, intravitreal endothelial growth factor inhibitor drugs (anti-VEGF), ocular steroids and so on [5, 6]. However, these available methods are not satisfactory. Simultaneously, lasers may cause permanent retinal damage [7], anti-VEGF therapy has a certain probability of causing endophthalmitis due to the need for multiple injections [8], and increased intraocular pressure is a common side effect of ocular steroids [9]. Therefore, systematic management of DR, including maintaining the stability of blood pressure, blood glucose and blood lipids, has gradually become a consensus in the ophthalmology community [10].

Statins are extensively used in clinical practice and can effectively lower serum low-density lipoprotein (LDL) cholesterol levels [11]. The inhibitory target of statins is 3-hydroxy-3-methylglutaryl-coenzyme A reductase (HMGCR), which has been verified enzyme to be involved in catalysing the production of cholesterol [12]. Since statins can significantly decrease cardiovascular and cerebrovascular disease risk in DM patients, statins are also recommended for lipid management in DM patients [13]. Notebly, the effectiveness of statins in treating DR is still controversial. According to basic research, statins are believed to be effective at reducing cholesterol accumulation and dissolving cholesterol crystals in the retina of DR patients to prevent endothelial disease [14]. A large cohort study also showed that statins significantly decreased the occurrence of DR and delayed its progression [15]. However, one meta-analysis was unable to determine the positive impact of statins on DR prevention and progression [16]. Another 17-year cohort study also revealed no protective effect of statins against DR [17]. The role of statins in DR urgently needs further exploration.

Owing to the true impact of statins on DR, these conditions cannot be determined by observational studies with diverse results. This study intends to adopt Mendelian randomization (MR) analysis and conduct a study based on data from the National Health and Nutrition Examination Survey (NHANES) to explore the effect of statins on DR. MR analysis evaluates the causal relationship between various risk factors and illness outcomes by using genetic variation as an instrumental variable (IV) [18, 19]. In contrast to traditional observational studies, MR analysis is able to avoid bias due to confounding factors and causal correlation occurrences that are not consistent with reality [20]. The second law of Mendel describes how genes combine randomly during meiosis and are unaffected by environmental factors. Accordingly, MR studies are unbiased, blind, and random [21]. Among the IVs used in current MR analyses, single nucleotide polymorphisms (SNPs) are the most widely used. Due to the fact that SNPs associated with exposure and outcome originate from diverse researches, SNPs are theoretically a perfect tool for estimating the causal effects of exposure on outcomes [22]. The National Health and Nutrition Examination Survey (NHANES) is a national project to promote human health based on a large amount of population interview data in the U.S. A cross-sectional study will also be performed utilizing the NHANES data to assess the accuracy of the MR analysis results and explore possible factors beyond the genetic level that may influence the relationship between statins and DR incidence.

## Methods

### Data sources of Mendelian randomization analysis and cross-sectional study

MR analysis was conducted with European IVs to minimize the confounding bias associated with racial factors. Expression quantitative trait loci (eQTL) data for HMGCR were obtained from eQTLGen Consortium (www.eqtlgen.org/), which included the upstream and downstream consequences of trait-related genetic variants from 31,684 blood samples [23]. GWAS data for DR (GWAS ID: finngen_R9_DM_RETINOPATHY_EXMORE, 14,584 cases and 176,010 controls), non-proliferative DR (NPDR) (GWAS ID: finngen_R9_H7_RETINOPATHYDIAB_BKG, 4011 cases and 344,569 controls), proliferative DR (PDR) (GWAS ID: finngen_R9_H7_RETINOPATHYDIAB_PROLIF, 2468 cases and 344,569 controls) and coronary atherosclerosis (GWAS ID: finngen_R9_I7_CORATHER, 47,550 cases and 313,400 controls) were obtained from FinnGen (freeze 9). LDL cholesterol-related GWAS data (GWAS ID: ieu-b-110, sample size: 440,546) originated from the Ieu Open Gwas Project, and the sample composition of the data was obtained from the UK Biobank [24]. No sample overlap was detected because the source populations of all the GWAS data included were diverse. The data for the cross-sectional study originated from the results of 10 cycles in the NHANES (sample size: 106,911, 2001–2020, www.cdc.gov/nchs/nhanes) and included demographic data, laboratory test results, and questionnaire results.

### Mendelian randomization analysis design and genetic instruments extraction

**Fig. 1:**
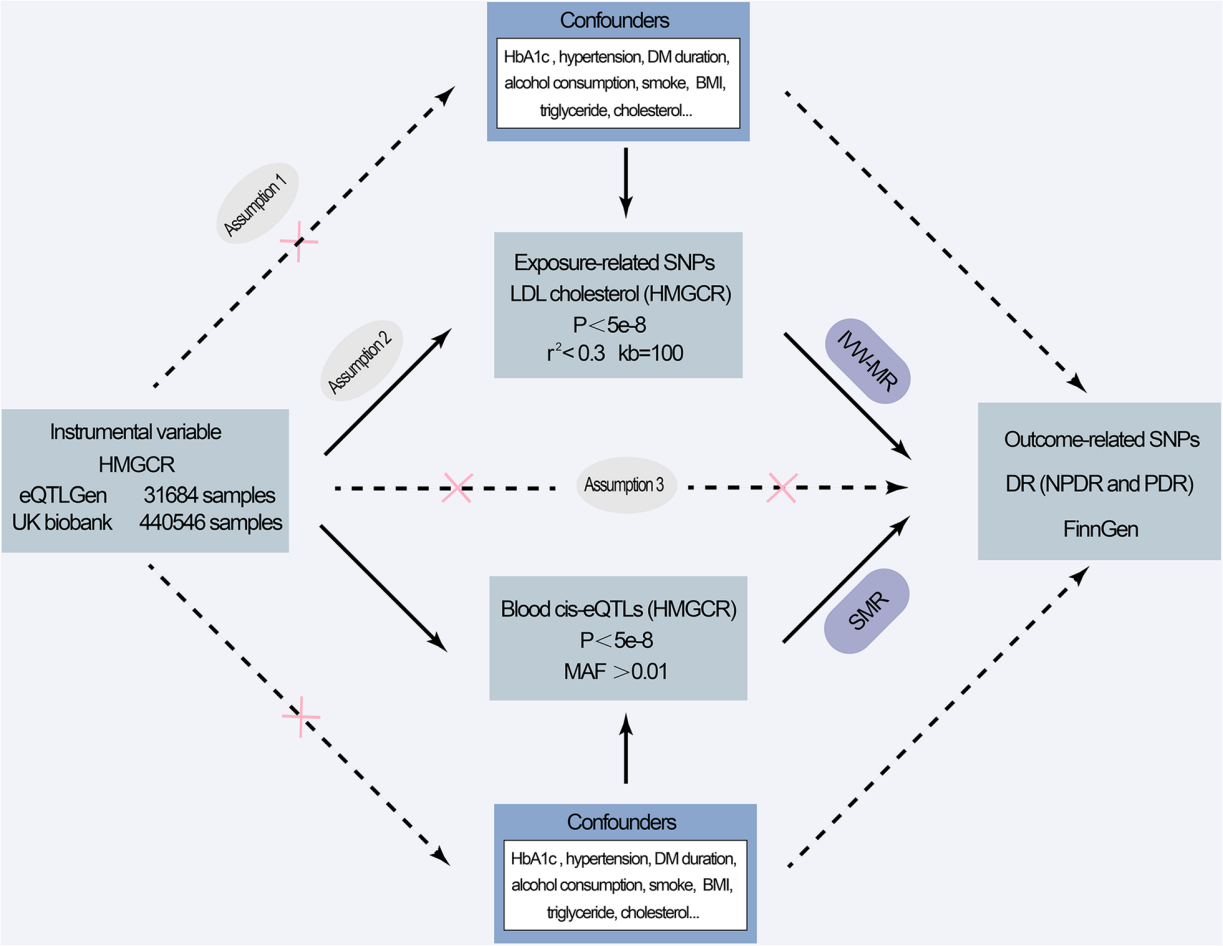
Schematic diagram of IVW-MR and SMR. DR: Diabetic retinopathy; NPDR: Non-proliferative diabetic retinopathy; PDR: Proliferative diabetic retinopathy; SNPs: Single nucleotide polymorphisms; IVW-MR: Inverse-variance-weighted mendelian randomization. SMR: Summary-data-based Mendelian randomization

The summary-data-based MR (SMR) method and inverse-variance-weighted MR (IVW-MR) method were utilized to infer the causal association between statins and DR. HMGCR, a statin-associated target gene, was used as a proxy for exposure. In terms of SMR, only cis-eQTLs within 1 Mb on both sides of HMGCR were used as the instrumental variable. We identified common SNPs in the blood that were significantly related to HMGCR by screening (minor allele frequency > 0.01, p < 5 × 10^−8^). In terms of IVW-MR, SNPs within 100 kb on both sides of the HMGCR gene locus were selected in the LDL cholesterol-related GWAS data as statin exposure proxies in this two-sample MR analysis. To maximize the strength of the extracted SNPs, we set r^2^ < 0.3 and kb = 100 during linkage disequilibrium analysis, considering only the SNPs that reached the GWAS threshold of statistical significance (p < 5 × 10^−8^) as IVs [25, 26]. At the same time, positive controls for HMGCR-related instrumental variables were also conducted. We used SMR methods to test the effect of HMGCR-associated SNPs on LDL cholesterol expression. Since LDL cholesterol is a recognized risk factor for coronary atherosclerosis [27, 28], IVW-MR was used to verify whether HMGCR-associated SNPs could further influence coronary atherosclerosis by affecting LDL cholesterol expression. This drug target-related MR study was conducted abiding by the Strengthening the Reporting of Observational Studies in Epidemiology Using Mendelian Randomization (STROBE-MR) guidelines; moreover, it is imperative for MR analysis to adhere to three crucial assumptions in the context of a two-sample MR study. These assumptions encompass the close association between instrumental variables (IVs) and the variables of interest, the independence of confounding factors from the relationship between exposure and outcome, and the assurance that IVs exert their influence on outcomes solely through the exposure variables. (Fig. 1) [29, 30]. The R^2^ (an indicator explaining the degree of exposure) [31] and F-statistic were calculated to measure the strength of the IVs (the calculation method is described in the Additional file 1). IVs with F-statistic < 10 were defined as weak IVs and were excluded [32]. When performing SMR analysis, β-exposure and standard error-exposure (Se-exposure) were used to estimate the F-statistic directly [33].

### Cross-sectional study design and participant screening

The DM diagnostic criteria are presented in the Additional file 1: Methods S1. Participants who answered “Yes” to the questionnaire question “Diabetes affected eyes/had retinopathy” were defined as DR patients. Statins use status were divided into three conditions: “taking statins” (Yes), “not taking statins” (No) and “taking other drugs” (Other). In addition to the demographic data including age, gender and race, we also included clinical covariates associated with DR, which contained DM duration, BMI, HbA1c, triglyceride, cholesterol, hypertension, smoke, and alcohol consumption. The races involved in this cross-sectional study included “Mexican American”, “Non-Hispanic Black”, “Non-Hispanic White”, “Other Hispanic”, “Non-Hispanic”, “non-Hispanic” and “Other race—including Multi-Racial”. The DM duration (years) was divided into 3 levels: “0–5”, “5–10” and “ > 10”. BMI was classified into 4 grades: “low weight” (< 18 kg/m^2^), “normal weight” (18–24 kg/m^2^), “overweight” (24–28 kg/m^2^), and “obese” (> 28 kg/m^2^). HbA1c concentrations (%) were divided into 2 grades, “ < 7” (good) and “ ≥ 7” (poor), to represent glycaemic control. Details on the hypertension definition and grading of smoking and alcohol consumption and age and race are provided in the Additional file 1: Methods S1. Both univariate analysis and multivariate analysis were carried out to evaluate the effect of statins use status on DR incidence. The univariate analysis result was regarded as a rough outcome reflecting the association between statins use status and DR incidence. The multivariate analysis included covariates with significant differences between DR patients and non-DR patients to exclude other factors that may bias the final results. Participants with missing medication records or incorrect records, non-diabetic patients, and pregnant participants were excluded.

### Statistical analysis

R (version 4.3.1) and SMR software (version 1.3.1) were utilized to conduct all MR analyses [22, 34, 35]. SPSS statistical software (version 23.0, Chicago, US) was used for the cross-sectional study. The fixed-effects IVW method was applied in scenarios devoid of heterogeneity [36]. In instances where heterogeneity was present, the multiplicative random-effects IVW model was employed [37]. Continuous variables are expressed as the means with 95% confidence intervals (CIs) (triglyceride and cholesterol, Gaussian distribution) or medians with interquartile ranges (age, non-Gaussian distribution). Categorical variables are expressed as frequencies (percentages). Odds ratios (ORs) with 95% CIs were calculated for the outcomes. To better represent the United States population, we weighted the values presented in the cross-sectional study. In the single-factor analysis model, we used the chi-square test to compare the DR group and non-DR group with "taking statins" as the reference group. In the multifactor analysis model, weighted multiple logistic regression analysis was adopted to evaluate the impact of statins use status on the risk of DR after adjusting covariates. The reference groups of each covariate are described in the Additional file 1: Methods S1. In sensitivity analyses for the IVW-MR method, as a first step, pleiotropy was assessed by performing the MR-Egger intercept test [38]. Subsequently, the presence of heterogeneity among causal estimates from various genetic variations was assessed through the application of Cochran’s Q test [39]. Additionally, leave-one-out analysis was conducted to evaluate the robustness of the results by excluding one SNP at a time [40]. Finally, outlier SNPs and horizontal pleiotropy were detected by applying MR pleiotropy residual sum and outlier (MR-PRESSO) and the outliers were further eliminated [34]. In sensitivity analyses for the SMR method, the heterogeneity in dependent instruments (HEIDI) test was applied to detect the linkage disequilibrium between the exposed variable and the outcome variable (p < 0.05 represents statistical significance) [41]. Due to the existence of multiple tests in this study, the Bonferroni correction method was employed to correct the significance threshold of the SMR and IVW-MR results. p < 0.017 (3 tests) represents strong significance, 0.017 ≤ p < 0.05 represents suggestive significance [42].

## Results

### Instrumental variable selection and participant screening

**Fig. 2:**
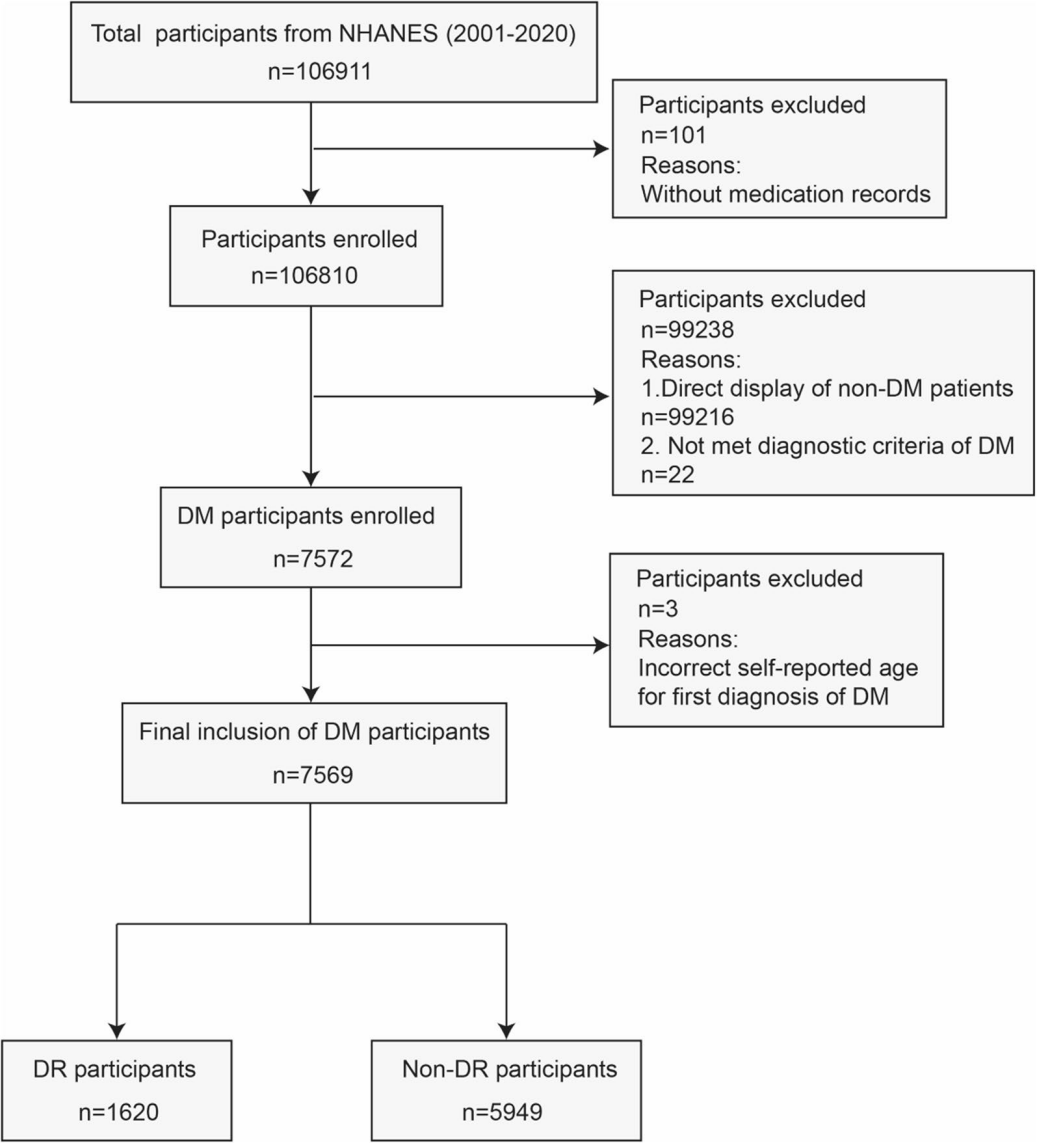
Flowchart presenting the process of participants screening in cross-sectional study. DM: Diabetes mellitus; DR: Diabetic retinopathy

In accordance with the strategy described above for extracting IVs, 921 eligible cis-eQTLs were extracted for SMR analysis, and the top SNP was rs6453133. Seventeen SNPs were eventually included in the IVW-MR analysis to investigate the potential causative relationships between statins and DR, NPDR, and PDR (Table 1). For every SNP considered, the F-statistic was significantly greater than 10, indicating that no weak instrument bias existed among the instrumental variables (Additional file 1: Table S1-S4). After screening eligible participants,7569 DM participants from our cross-sectional study based on the NHANES were eventually included in our analysis (Fig. 2).

**Table 1:** Results of Cochran’s Q test, MR-Egger-intercept test, MR-PRESSO and HEIDI test for MR analyses of causal relations between statins and DR

Outcome	No. (1)	No. (2)	Cochran’s Q test		MR-Egger intercept test		MR-PRESSO	HEIDI test	
			Q statistic	P-value	Intercept	P-value	P-value of global test	P-value	nSNP
DR	921	17	14.26	0.58	0.023	0.23	0.66	0.59	20
NPDR		17	5.54	0.99	0.023	0.44	0.99	0.46	20
PDR		17	18.58	0.29	0.044	0.28	0.36	0.31	20

DR: Diabetic retinopathy; NPDR: Non-proliferative diabetic retinopathy; PDR: Proliferative diabetic retinopathy; No. (1): Numbers of eligible cis-eQTLs (expression quantitative trait loci studies) of HMGCR (3-hydroxy-3-methylglutaryl-coenzyme A reductase); No. (2): Numbers of genetic instruments not containing palindromic sequences or not being the outliers. MR: Mendelian randomization; MR-PRESSO: Mendelian randomization pleiotropy residual sum and outlier; HEIDI: Heterogeneity in dependent instruments, nSNP: Numbers of single nucleotide polymorphism

### MR analysis of HMGCR expression with risk of diabetic retinopathy

**Fig. 3:**
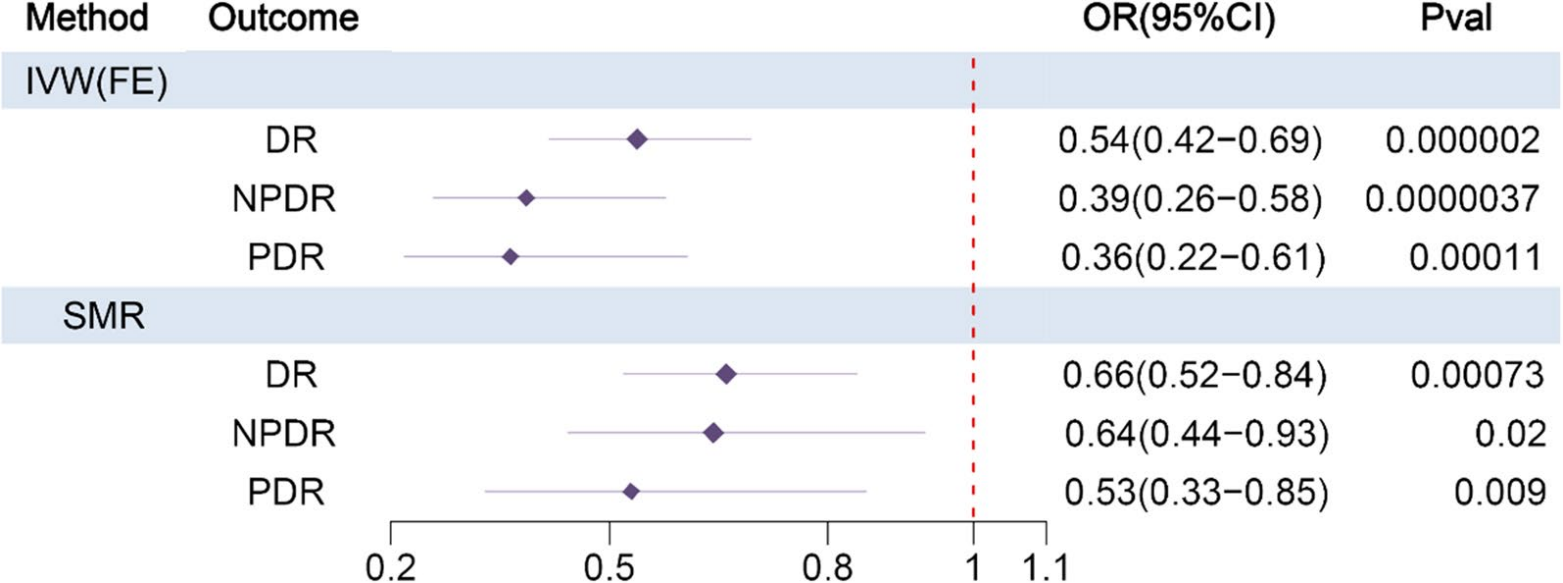
The forest plot existing causal effect of HMGCR expression on DR. HMGCR: 3-hydroxy-3-methylglutaryl-coenzyme A reductase; IVW (FE): Fixed effects inverse-variance-weighted model; SMR: Summary-data-based mendelian randomization; DR: Diabetic retinopathy; NPDR: Non-proliferative diabetic retinopathy; PDR: Proliferative diabetic retinopathy

Both SMR analysis (OR = 0.66, 95% CI [0.52, 0.84], p = 0.00073) and IVW-MR analysis (OR = 0.54, 95% CI [0.42, 0.69], p = 0.000002) of HMGCR-DR revealed that upregulated HMGCR expression was causally associated with a significantly decreased risk of DR. DR was further classified into two subgroups (NPDR and PDR) based on the severity of the disease, and a causal association between HMGCR expression and NPDR or PDR was also detected. The results of SMR analysis and IVW-MR analysis for HMGCR-NPDR and HMGCR-PDR were consistent with the results of HMGCR-DR, and upregulated expression of HMGCR was causally associated with a significantly decreased risk of NPDR and PDR (Fig. 3). Positive control results showed that upregulated HMGCR gene expression significantly increased the expression of LDL cholesterol (Additional file 1: Table S5), and elevated HMGCR gene expression in GWAS data related to LDL cholesterol demonstrated a causal association with a notably heightened risk of coronary atherosclerosis (Additional file 1: Table S6).

### Association of statins use status with the risk of diabetic retinopathy

The baseline data of the included participants exhibited significant differences in HbA1c levels, DM duration, hypertension incidence, alcohol consumption and statins use status between the DR and non-DR groups, and these factors were included in the multivariate analysis model for further calculation (Table 2). The results of the single-factor analysis preliminarily revealed a notable decrease in the likelihood of developing DR among participants who did not take statins (OR = 0.36, 95% CI [0.26, 0.50], p < 0.001). The results of weighted multiple logistic regression analysis revealed that not taking statins was significantly associated with a decreased risk of DR after adjusting for other covariates (OR = 0.54, 95% CI [0.37, 0.79], p = 0.001). To test the stability of our model, two covariables (age and race) whose baseline data comparison results were close to the threshold were also added to generate another model for analysis, and no significant changes were found in the results (Table 3).

**Table 2:** Baseline characteristics of enrolled DR and non-DR participants

	Total (N = 7569)	non-DR (N = 5949)	DR (N = 1620)	P val
Age (years)	63 (54,72)	63 (53,72)	64 (55,73)	0.055
Gender				0.776
Female		2888 (48.55%)	780 (48.15%)	
Male		3061 (51.45%)	840 (51.85%)	
Race				0.058
Mexican American	1361 (17.98%)	1069 (17.97%)	292 (18.02%)	
Non-Hispanic Black	2063 (27.26%)	1611 (27.08%)	452 (27.90%)	
Non-Hispanic White	2663 (35.18%)	2137 (35.92%)	526 (32.47%)	
Other Hispanic	685 (9.05%)	520 (8.74%)	165 (10.19%)	
Other race—including multi-racial	797 (10.53%)	612 (10.29%)	185 (11.42%)	
BMI(kg/m^2^)				0.128
Low weight	30 (0.4%)	25 (0.5%)	5 (0.3%)	
Normal weight	974 (14.0%)	747 (13.6%)	227 (15.5%)	
Overweight	2091 (30.0%)	1679 (30.5%)	412 (28.1%)	
Obese	3877 (55.6%)	3054 (55.5%)	823 (56.1%)	
HbA1c (%)				< 0.001
< 7.0	3629 (52.8%)	3001 (55.5%)	628 (43.1%)	
≥ 7.0	3238 (47.2%)	2410 (44.5%)	828 (56.9%)	
Triglyceride (mg/dl)	148 (101,220)	148 (101,220)	148 (99,221)	0.966
cholesterol (mg/dl)	176 (149,208)	176 (150,208)	174 (147,209)	0.615
Hypertension				< 0.001
No	2028 (26.9%)	1661 (28.0%)	367 (22.7%)	
Yes	5525 (73.1%)	4275 (72.0%)	1250 (77.3%)	
Smoke				0.165
Never	3766 (50.2%)	2938 (49.9%)	828 (51.2%)	
Former	2555 (34.1%)	1997 (33.9%)	558 (34.5%)	
Now	1182 (15.8%)	952 (16.2%)	230 (14.2%)	
Alcohol consumption				< 0.001
Never	1132 (19.0%)	868 (18.5%)	264 (21.2%)	
Former	1524 (25.6%)	1157 (24.6%)	367 (29.5%)	
Mild	1975 (33.2%)	1600 (34.0%)	375 (30.1%)	
Moderate	597 (10.0%)	500 (10.6%)	97 (7.8%)	
Heavy	719 (12.1%)	577(12.3%)	142 (11.4%)	
DM duration (years)				< 0.001
0–5	2484 (33.6%)	2168 (37.5%)	316 (19.7%)	
5–10	1593 (21.6%)	1310 (22.6%)	283 (17.7%)	
> 10	3312 (44.8%)	2309 (39.9%)	1003 (62.6%)	
Statin use status				< 0.001
Not taking statins	414 (5.5%)	373(6.3%)	41 (2.5%)	
Taking other drugs	3278 (43.3%)	2604 (43.8%)	674 (41.6%)	
Taking statins	3877 (51.2%)	2972 (50.0%)	905 (55.9%)

**Table 3:** Association between statins use status and risk of DR

Outcome	Exposure	Type of medication	Model 1			Model 2			Model 3		
			OR	95%CI	P val	OR	95%CI	Pval	OR	95%CI	P val
DR	Statin use status	Taking statins	Refs.			Refs.			Refs.		
		Taking other drugs	0.85	(0.76,0.95)	0.005	0.94	(0.82,1.08)	0.54	0.92	(0.81,1.07)	0.29
		Not taking statins	0.36	(0.26,0.50)	< 0.001	0.54	(0.37,0.79)	0.001	0.51	(0.35,0.75)	0.001

DR: Diabetic retinopathy; Model 1: Univariate analysis model; Model 2: Multivariate analysis model adjusted for HbA1c levels, hypertension prevalence, DM duration and alcohol consumption; Model 3: Multivariate analysis model adjusted for HbA1c levels, hypertension prevalence, DM duration, alcohol consumption, age and race

### Sensitivity analysis for MR analysis

The results of the HEIDI test revealed no linkage disequilibrium between the exposed variable and the outcome variable in any of the SMR analyses. No pleiotropy or heterogeneity existed in our MR analyses based on the results of the MR-Egger-intercept test or Cochran's Q test. The overall findings from all analyses remained stable, with no significant changes observed when individual SNPs were systematically excluded, as evidenced by the leave-one-out analysis results (Additional file 1: Fig. S1). MR-PRESSO detects one outlying SNP causing horizontal pleiotropy effects in HMGCR-coronary atherosclerosis IVW-MR analysis. Global test did not reveal any possible level of pleiotropy. The sensitivity results described above fully indicated that all the MR analysis results were reliable and stable (Table 1).

## Discussion

The high incidence of DM worldwide has led to a surge in DR patients, and DR has emerged as the primary cause of visual impairment among the global working-age population [43]. Dyslipidaemia is a recognized risk factor for DR, so it is critical to reasonably control blood lipid levels [44, 45]. In particular, hypercholesterolemia has been demonstrated to be associated with hard exudates in the retina of DR patients [46]. Elevated LDL cholesterol can be modified to form advanced glycation end product LDL and oxidized LDL to cause microvascular damage and further aggravate DR progression [47]. Statins, as classic lipid-lowering drugs, can effectively downregulate the level of serum LDL cholesterol [11]. Several studies have suggested that statins has a positive impact on DR, for example, several basic experiments in animals have confirmed that statins can improve DR by inhibiting neovascularization [48, 49], but the level of evidence supporting these results is still low in the field of evidence-based medicine. Although a meta-analysis comprising six cohort studies supported the role of statins in improving DR and its subtypes [50], most of these six studies failed to include or only partially included the factors (cholesterol, triglycerides, HbA1c, DM duration, smoking, hypertension, BMI, etc.) that may affect the incidence of DR among the included participants, which may have led to a large bias in the final results. Therefore, the true efficacy of statins in treating DR remains unclear.

This study was the first to explore the causal connection between statins and DR from a genetic perspective and to use big data-based MR analysis, which can effectively eliminate the potential confounding bias existing in previous observational epidemiological studies. In conducting this MR analysis, MR analysis was conducted strictly according to the three core assumptions to minimize confounding factors and improve the reliability of the results. Since the gene target of statins inhibition is HMGCR [12], we used HMGCR-related instrumental variables as an exposure proxy for statins to detect the causal association between statins and DR. Our SMR analysis and IVW-MR analysis provided robust support for a shared genetic association between statins and DR, revealing that upregulated expression of HMGCR significantly decreases the risk of DR, on the other hand, statins can causally increase the risk of DR by inhibiting HMGCR expression. On the basis of our subgroup analysis, the causal effect of statins on DR was not associated with the severity of the illness, and the upregulation of HMGCR expression had a protective effect on both NPDR and PDR. After correcting the p-value via the Bonferroni correction method, all the results were strongly significant other than the results of SMR for HMGCR-NPDR (P = 0.02) was suggestive significant. In terms of sensitivity analysis, the results of sensitivity analysis for IVW-MR did not reveal heterogeneity or horizontal pleiotropy, and the sensitivity analysis for SMR did not indicate the existence of linkage disequilibrium, which fully demonstrates the reliability of our outcomes. Moreover, the results of a positive control test further proved that the instrumental variables of HMGCR used in this study, including eQTL of HMGCR and the HMGCR gene locus extracted from the LDL cholesterol-related GWAS data, were reliable as exposure proxies.

Although MR analysis can confirm the harmful causal effect of statins on DR, the influence of lipid-lowering agents on the overall condition of DR patients cannot be ruled out. To further verify the results of the MR analysis, a cross-sectional study based on NHANES involving a large number of U.S. population was also conducted, and the conclusions of both the univariate analysis model and the multifactor analysis model supported the association between statins use and increased risk of DR. Noteworthily, there were no significant differences in blood lipid markers (triglycerides and cholesterol) between participants in the "taking statins" group and the "not taking statins" group. The baseline information is presented in Table 2, which precluded the potential influence of human lipid markers in comparing the incidence of retinopathy in DM patients in the "taking statins" and "not taking statins" groups. This is precisely the significance of this cross-sectional study. Moreover, the specific mechanism of action of circulating lipids in microangiopathy is unclear, especially in DR [51]. A meta-analysis based on the MR principle suggested that statins may promote insulin resistance through weight gain and thus increase blood glucose, which could be explained in part by inhibiting HMGCR [52]. The increase in blood glucose caused by statins may be a potential cause for exacerbating the risk and progression of DR, however, these studies included few instrumental variables, and the blood glucose data of the included population did not include fasting blood glucose values. A comparative study based on pericyte lines and C57B1/6 mice demonstrated that HMGCR inhibitors promote DR microangiopathy by promoting pericyte apoptosis [53]. HMGCR inhibition can also reduce the expression of cdc42 in endothelial cells to destabilize the blood vessels and increase vascular permeability [54]. M J Liinamaa et al. reported that using statins can significantly increase VEGF concentrations in the vitreous body of PDR patients [55]. VEGF can promote the progression of DR by disrupting the blood-retinal barrier and causing neovascularization [56]. Therefore, the role of statins in DR may be inspired by exploring the association between HMGCR and microvascular lesions in DR, which requires additional experimental evidence to further prove its authenticity.

This study was the first to show that statins may possess an adverse effect on DR, and can overcome the ethical issues that ordinary randomized controlled trials may face. MR analysis essentially yields a natural randomized controlled trial (RCT), and the GWAS and eQTL data used in this study were also extracted from the latest versions to date and cover the largest number of European. Therefore, to ascertain the causal relationship between statins and DR, the MR analysis method employed in this study remains the most effective approach. Notably, this MR analysis only extracted HMGCR-related eQTL and instrumental variables of HMGCR in LDL cholesterol GWAS data for MR analysis, and our cross-sectional study concentrated only on the effect of statins use status on the risk of developing DR, while other lipid-lowering drugs and related genes were not discussed in this study. Thus, the conclusion of this study does not deny the beneficial effect of cholesterol lowering agents on DR. Our findings provide constructive advice for long-term lipid management in patients with DR, suggesting that the use of statins in DR patients should be considered more carefully.

Nevertheless, the following limitations still exist in the current study: (1) Owing to restricted GWAS data mining, we did not further use the eQTL of other human tissues related to lipid metabolism for SMR analysis in this study. Human tissues related to lipid metabolism include adipose tissue and non-adipose tissue, and non-adipose tissue is mainly represented by liver and muscle [57]; (2) The results of Bonferroni correction indicate that the evidence level of SMR results based on HMGCR-NPDR is suggestive significant, which makes the conclusion that statins increase the risk of NPDR possible to be false positive; (3) As this study only used GWAS data from the European population, it cannot be a good representation of other ethnicities or races around the world; (4) While substantial evidence supports the link between serum cholesterol and DR [46, 58], some studies suggest that statins primarily influence DR through the leakage and clearance of lipids in the retina [59]. Given the lack of available eQTL data from retinal tissue, and the HMGCR-related eQTL data utilized in this study are derived from blood samples, acquiring HMGCR-related eQTL data from retinal tissue for future research could substantially enhance the validity of this study's findings; (5) In cross-sectional study, although we have tried to expand the inclusion of participants from NHANES, the number of participants involved in the analysis is still limited, which might lead to unnecessary bias in results calculation. Self-reported recall bias for DR diagnosis could not also be completely ruled out; and (6) In the multiple regression analysis, some covariates might be not taken into account. Hence, the findings of this study warrant further validation through multi-center epidemiological research and genetic engineering experiments, employing a larger sample size and diverse populations.

## Conclusions

With the use of MR analysis, this study first explored the causal impact of statins on DR from a genetic perspective, and the results were also validated by a cross-sectional study based on the NHANES. The outcomes of the present research confirmed a causal association between statins use and a significantly heightened risk of DR. The accurateness and validity of the study findings necessitate further verification through additional basic and clinical studies exploring the mechanisms and impact of statins on DR.

### Supplementary Information

**Additional file 1:** **Methods S1.** **Figure S1.** Results of leave-one-out analysis. **A** Leave-one-out analysis for analyzing the causal association between statins and DR; **B** Leave-one-out analysis for analyzing the causal association between statins and NPDR; **C** Leave-one-out analysis for analyzing the causal association between statins and PDR; **D** Leave-one-out analysis for analyzing the causal association between statins and coronary atherosclerosis. DR: Diabetic retinopathy; NPDR: Non-proliferative diabetic retinopathy; PDR: Proliferative diabetic retinopathy. **Table S1.** MR analysis data of the causal effect of statins on DR. **Table S2.** MR analysis data of the causal effect of statins on NPDR. **Table S3.** MR analysis data of the causal effect of statins on PDR. **Table S4.** MR analysis data of the causal effect of statins on coronary atherosclerosis. **Table S5.** SMR association between HMGCR (ProbeID: ENSG00000113161) expression and diverse outcomes. **Table S6.** Results of Cochran’s Q test, MR-Egger-intercept test and MR-PRESSO for MR analyses of causal relations between statins and coronary atherosclerosis.

## Data Availability

The data presented in this study are available in article and supplementary materials.
