# Supplementary material for: Statins as a risk factor for diabetic retinopathy: a Mendelian randomization and cross-sectional observational study
Source: J Transl Med. 2024 Mar 22;22:298. doi: 10.1186/s12967-024-05097-8 (PMC10958895; doi:10.1186/s12967-024-05097-8)
Supplement: Supplementary file 1 — Additional file 1: Methods S1. Figure S1. Results of leave-one-out analysis. A Leave-one-out analysis for analyzing the causal association between statins and DR; B Leave-one-out analysis for analyzing the causal association between statins and NPDR; C Leave-one-out analysis for analyzing the causal association between statins and PDR; D Leave-one-out analysis for analyzing the causal association between statins and coronary atherosclerosis. DR: Diabetic retinopathy; NPDR: Non-proliferative diabetic retinopathy; PDR: Proliferative diabetic retinopathy. Table S1. MR analysis data of the causal effect of statins on DR. Table S2. MR analysis data of the causal effect of statins on NPDR. Table S3. MR analysis data of the causal effect of statins on PDR. Table S4. MR analysis data of the causal effect of statins on coronary atherosclerosis. Table S5. SMR association between HMGCR (ProbeID: ENSG00000113161) expression and diverse outcomes. Table S6. Results of Cochran’s Q test, MR-Egger-intercept test and MR-PRESSO for MR analyses of causal relations between statins and coronary atherosclerosis. [file 12967_2024_5097_MOESM1_ESM.docx]

**Supplement**

**Table of contents**

1. Statement of methods……………………………………………………………………………............................2
2. Supplementary figure ……………………………………………...……………………………............................5
3. Supplementary tables (1-6)…………………………………………………………………….............................6

**Supplementary Methods**

**Data Synthesis：**

Mendelian randomization analysis: Beta value, Standard error (Se), Effect allele frequency (EAF) and P value of exposure-related SNPs and outcome-related SNPs were from eQTLGen Consortium ([www.eqtlgen.org/](https://www.eqtlgen.org/)), Finngen GWAS database (https://r9.finngen.fi/) and Ieu Open Gwas Project (https://gwas.mrcieu.ac.uk). R² value was calculated by formula: R²=2×beta²×EAF×(1-EAF)/(2×beta²×EAF×(1-EAF)+Se²×2×N×EAF×(1-EAF)) [1], all data in the formula are derived from exposed variables, N represents the number of samples of GWAS exposed studies. F- statistic was calculated on the basis of R² value by formula F=((N-k-1)/k) × (R²/(1- R²)) [2], N represents the number of samples of GWAS exposed studies, k represents the number of instrumental variables. If the data source did not provide Effect allele frequency (EAF), we directly used β-exposure and standard error-exposure (Se-exposure) to estimate the F-statistic (F=(β-exposure) ²/ (Se-exposure) ²) [3]. We organized the IVW-MR analysis data of the causal relationship between statins and DR, NPDR, PDR or coronary atherosclerosis into eTable1-4. SNPs containing palindromic sequences will also be further excluded when harmonising the exposure-releated SNPs and outcome-related SNPs. P < 0.05 was considered statistically significant in sensitivity analyses.

Cross-sectional study: According to the NHANES database, the statins referred to in this study include simvastatin, rosuvastatin, pravastatin, lovastatin, fluvastatin, simvastatin, atorvastatin.

**Diagnostic criteria of diabetes mellitus:**

1. doctor told you have diabetes,

2. glycohemoglobin HbA1c(%) >= 6.5,

3. fasting glucose (mmol/l) >= 7.0,

4. random blood glucose (mmolll) >= 11.1,

5. two-hour OGTT blood glucose (mmol/l) >= 11.1,

6. Use of diabetes medication or insulin.

DM: diabetes mellitus; IFG: Impaired Fasting Glycaemia; IGT: Impaired Glucose Tolerance

**Hypertension definition:**

Hypertension was defned as a blood pressure reading over 140/90 mmHg. Average blood presure was calculated by the following protocol: The diastolic reading with zero is not used to calculate the diastolic average. lf all diastolic readings were zero, then the average would be zero. lf only one blood pressure reading was obtained, that reading is the average. lf there is more than one blood pressure reading, the first reading is always excluded from the average.

Group “yes” was regarded as the reference group in the multivariate analysis model.

**Grading of smoking：**

never: smoked less than 100 cigarettes in life;

former: smoked more than 100 cigarettes in life and smoke not at all now;

now: smoked moth than 100 cigarettes in life and smoke some days or every day.

**Grading of alcohol consumption：**

never: had <12 drinks in lifetime;

Former: had ≥12 drinks in 1 year and did not drink last year, or did not drink last year but drank ≥12 drinks in lifetime;

mild = had ≥1 drinks per day for females, ≥2 drinks per day for males;

moderate = had ≥2 drinks per day for females, ≥3 drinks per day for males, or binge drinking ≥2 days per month;

heavy = had ≥3 drinks per day for females, ≥4 drinks per day for males, or binge drinking on 5 or more days per month;

binge drinking: had ≥4 drinks on same occasion for females, ≥5 drinks on same occasion for males [4, 5];

Group “never” was regarded as the reference group in the multivariate analysis model.

**DM duration (years)：**

Group “5-10” was regarded as the reference group in the multivariate analysis model.

**HbA1c concentrations (%):**

Group “＜7” was regarded as the reference group in the multivariate analysis model.

**Statins use status:**

“taking statins” represents “participants who were taking statins only”,

“taking other drugs” represents “participants who were taking other types of medication besides statins”,

“not taking statins” represents “participants who were not taking drugs at all”,

Group “taking statins” was regarded as the reference group in the multivariate analysis model.

**Age and race (model 3):**

Since the age distribution of the enrolled participants was non-Gaussian, when age was included in the multivariate analysis model, we would use the four-classification method to divide age into four levels of “0-25%”, “25-50%”, “50-75%” and “75-100%” when age was included in the multivariate analysis model. Group “75-100%” was used as the reference group in the multivariate analysis model. Group “Other Race - Including Multi-Racial” was regarded as the reference group in the multivariate analysis model.

**Reference:**

1. Palmer, T.M., et al., *Using multiple genetic variants as instrumental variables for modifiable risk factors.* Stat Methods Med Res, 2012. **21**(3): p. 223-42.

2. Burgess, S. and S.G. Thompson, *Avoiding bias from weak instruments in Mendelian randomization studies.* Int J Epidemiol, 2011. **40**(3): p. 755-64.

3. Li, S., et al., *Ankylosing spondylitis and glaucoma in European population: A Mendelian randomization study.* Front Immunol, 2023. **14**: p. 1120742.

4. Rattan, P., et al., *Inverse Association of Telomere Length With Liver Disease and Mortality in the US Population.* Hepatol Commun, 2022. **6**(2): p. 399-410.

5. Hicks, C.W., et al., *Peripheral Neuropathy and All-Cause and Cardiovascular Mortality in U.S. Adults : A Prospective Cohort Study.* Ann Intern Med, 2021. **174**(2): p. 167-174.

**Figure S1** Results of leave-one-out analysis. (A) Leave-one-out analysis for analyzing the causal association between statins and DR; (B) Leave-one-out analysis for analyzing the causal association between statins and NPDR; (C) Leave-one-out analysis for analyzing the causal association between statins and PDR; (D) Leave-one-out analysis for analyzing the causal association between statins and coronary atherosclerosis. DR: Diabetic retinopathy; NPDR: Non-proliferative diabetic retinopathy; PDR: Proliferative diabetic retinopathy.


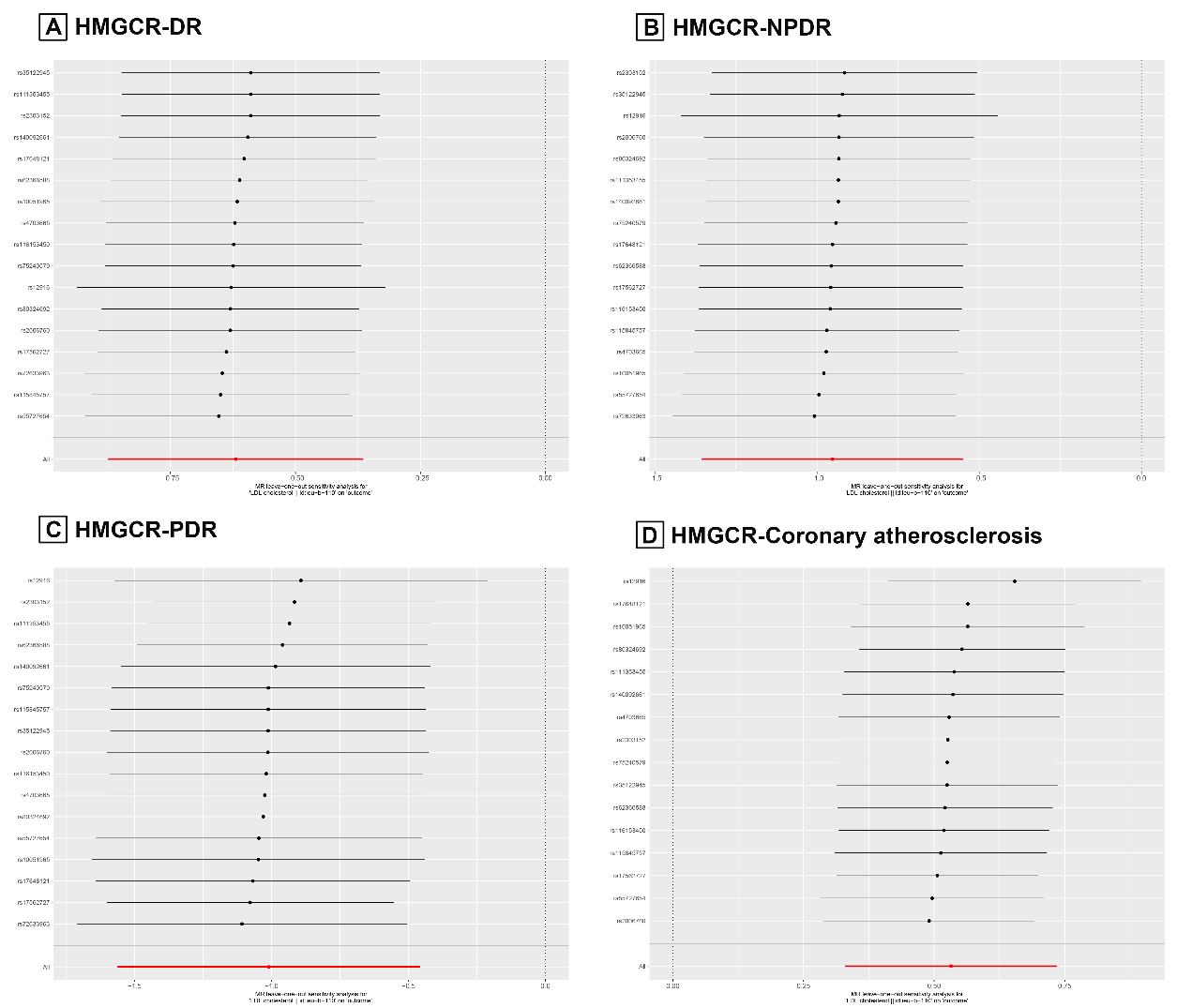


**Table S1** MR analysis data of the causal effect of statins on DR.

Abbreviations: SNP: single nucleotide polymorphism; MR: Mendelian randomization; Chr: chromosome; A1: Effect allele; A2: Other allele; Eaf: Effect allele frequency; HMGCR: 3-hydroxy-3-methylglutaryl-coenzyme A reductase; LDL: low density lipoprotein; DR: Diabetic retinopathy; Beta: Beta-coefficient; Se: Standard error; Pval: p-value; F: F-statistic.

**Table S2** MR analysis data of the causal effect of statins on NPDR.

Abbreviations: SNP: single nucleotide polymorphism; MR: Mendelian randomization; Chr: chromosome; A1: Effect allele; A2: Other allele; Eaf: Effect allele frequency; HMGCR: 3-hydroxy-3-methylglutaryl-coenzyme A reductase; LDL: low density lipoprotein; NPDR: Non- proliferative diabetic retinopathy; Beta: Beta-coefficient; Se: Standard error; Pval: p-value; F: F-statistic.

**Table S3** MR analysis data of the causal effect of statins on PDR.

Abbreviations: SNP: single nucleotide polymorphism; MR: Mendelian randomization; Chr: chromosome; A1: Effect allele; A2: Other allele; Eaf: Effect allele frequency; HMGCR: 3-hydroxy-3-methylglutaryl-coenzyme A reductase; LDL: low density lipoprotein; PDR: Proliferative diabetic retinopathy; Beta: Beta-coefficient; Se: Standard error; Pval: p-value; F: F-statistic.

**Table S4** MR analysis data of the causal effect of statins on coronary atherosclerosis.

Abbreviations: SNP: single nucleotide polymorphism; MR: Mendelian randomization; Chr: chromosome; A1: Effect allele; A2: Other allele; Eaf: Effect allele frequency; HMGCR: 3-hydroxy-3-methylglutaryl-coenzyme A reductase; LDL: low density lipoprotein; CA: Coronary atherosclerosis; Beta: Beta-coefficient; Se: Standard error; Pval: p-value; F: F-statistic.

**Table S5** SMR association between HMGCR (ProbeID: ENSG00000113161) expression and diverse outcomes.

Abbreviations: SMR: summary-data-based mendelian randomization; SNP: single nucleotide polymorphism; HMGCR: 3-hydroxy-3-methylglutaryl-coenzyme A reductase; LDL: low density lipoprotein; Chr: chromosome; BP: Base pair; EAF: Effect allele frequency; eQTL: Expression quantitative trait loci; DR: Diabetic retinopathy; NPDR: Non-proliferative diabetic retinopathy; PDR: Proliferative diabetic retinopathy; Beta: Beta-coefficient; Se: Standard error; Pval: p-value; F: F-statistic.

**Table S6** Results of Cochran's Q test, MR-Egger-intercept test and MR-PRESSO for MR analyses of causal relations between statins and coronary atherosclerosis.

Abbreviations: CA: Coronary atherosclerosis; No. (1): Numbers of genetic instruments after clumping; No. (2): Numbers of genetic instruments not containing palindromic sequences or not being the outliers. MR: Mendelian randomization; IVW (MRE): Multiplicative random-effect inverse-variance-weighted model; MRE: MR-PRESSO: Mendelian randomization pleiotropy residual sum and outlier.
